# Effects of *Saccharomyces boulardii* Supplementation on Nutritional Status, Fecal Parameters, Microbiota, and Mycobiota in Breeding Adult Dogs

**DOI:** 10.3390/vetsci9080389

**Published:** 2022-07-28

**Authors:** Giorgia Meineri, Elisa Martello, David Atuahene, Silvia Miretti, Bruno Stefanon, Misa Sandri, Ilaria Biasato, Maria Rita Corvaglia, Ilario Ferrocino, Luca Simone Cocolin

**Affiliations:** 1 Department of Veterinary Sciences, School of Agriculture and Veterinary Medicine, University of Turin, 10095 Grugliasco, Italy; giorgia.meineri@unito.it (G.M.); david.atuahene@unito.it (D.A.); silvia.miretti@unito.it (S.M.); 2 Centre for Evidence Based Healthcare, School of Medicine, University of Nottingham, Nottingham NG5 1PB, UK; 3 Department of Agrifood, Environmental and Animal Science, University of Udine, 33100 Udine, Italy; bruno.stefanon@uniud.it (B.S.); misa.sandri@nutrigenefood.com (M.S.); 4 Department of Agricultural, Forest and Food Sciences, University of Turin, 10095 Grugliasco, Italy; ilaria.biasato@unito.it (I.B.); mariarita.corvaglia@unito.it (M.R.C.); ilario.ferrocino@unito.it (I.F.); lucasimone.cocolin@unito.it (L.S.C.)

**Keywords:** supplement, alternative medicine, pet, Italy

## Abstract

**Simple Summary:**

The aim of this study was to evaluate the effect of the administration of *Saccharomyces boulardii* on the nutritional, immunological, inflammatory, stress status, and the gut composition in 25 healthy adult American Staffordshire Terrier dogs. Supplementation with *S. boulardii* significantly improved the intestinal status and induced a reduction of stress, a common condition affecting animals managed in a breeding environment.

**Abstract:**

The aim of this study was to evaluate the effect of the administration of *Saccharomyces boulardii* on the nutritional, immunological, inflammatory, and stress status and on the composition of the gut microbiota and mycobiota in healthy adult dogs. A total of 25 American Staffordshire Terrier dogs were selected and randomly assigned to two groups: control (CTR, n = 12) and treated (TRT, n = 13) groups. No significant differences were found between the two groups regarding body weight, body condition score, and fecal score. No significant differences in microbiota/mycobiota, short chain fatty acids, indole/skatole, histamine, zonulin, or lactoferrin were detected. Indeed, supplementation with *S. boulardii* significantly decreased fecal calprotectin Immunoglobulin A, indicating an improvement in the gut well-being. Interestingly, fecal cortisol significantly decreased in dogs belonging to the TRT group compared to the CTR, suggesting both an improvement of the intestinal status and a reduction of stress, a common condition affecting animals managed in a breeding environment.

## 1. Introduction

Gut microbiota have several roles in maintaining the animal health status, including the defense against pathogens, the development of a healthy intestinal epithelium and immune system, absorption, and the metabolism of ingested nutrients [1,2]. The “healthy gut” is linked to the well-being of the host. For example, the gut microbiota are essential for maintaining the homeostasis of the host by affecting the functions of the brain, liver, heart, kidney, immune system, and the metabolism of adipose tissue [3,4,5]. Dysbiosis is sued by microbes’ unbalance in the gastrointestinal (GI) tract, inducing a negative impact on health. Dysbiosis in healthy adult dogs is often associated with aging but can also be observed in animals living in stabled conditions. Dogs that live in breeding conditions can be much more exposed than companion dogs to chronic stress related to confined environments with spatial restrictions, lack of environmental stimuli, and imposed social interactions [6]. Therefore, due to the well-known link between the gut and brain, chronic stress can result in dysbiotic conditions (i.e., diarrhea) and greater susceptibility to GI disorders. Treatments commonly include the use of antibiotics increasing the risk of antimicrobial resistance [7,8,9]. Optimizing intestinal eubiosis is essential for the well-being and psycho-physical balance of animals. Probiotics are largely used to maintain gastrointestinal health. Probiotics are defined as “live microorganisms” which confer positive effects on the host’s health when administered at the correct dosage [10]. They can promote the GI health and mitigate dysbiosis due to stress stimuli in farm animals [11]. Studies reported the benefits of using *Saccharomyces boulardii* [12,13,14] as a probiotic. Specifically, it supports the barrier function and the regeneration of intestinal tissue; it is a valid alternative to the use of antimicrobial molecules in counteracting dysbiosis [15,16].

The aim of this study was to show the effects of *S. boulardii* in breeding dogs on selected nutritional parameters and on regulation of inflammatory, immunological, and stress indicators. In addition, the composition of the intestinal microbiota and mycobiota was evaluated.

## 2. Materials and Methods

### 2.1. Animals and Study Design

In this study, American Staffordshire Terrier dogs were selected from an ENCI (Ente Nazionale Cinofilia Italiana) registered breeder located in the north of Italy. The dog breeder was informed of the purpose and design of the study and signed a written informed consent form. The study was conducted in compliance with the guidelines of the Ministry of Health for the care and use of animals (DL 4 March 2014 n.26 and DL 27 January 1992 n.116) and EU (Directive 86/609/EEC); the use of supplements was governed by Regulation (EC) no. 767/2009. The study was approved by the University of Turin with protocol number 156895, 14.04.2020.

The age of the dogs ranged from 2 to 8 years (mean 5.69 ± 1.8 SD TRT group and mean 3.67 ± 1.83 SD CTR group). A total of 8 dogs were males (n = 4 TRT and n = 4 CTR), and 17 were females (n = 9 TRT and n = 8 CTR).

At the beginning of the study, the veterinarian checked the health status of the animals through a general physical examination and a copromicroscopic examination of the feces. All the recruited animals were healthy with no underlined conditions. A total of 25 dogs were kept in boxes (2/3 per box). The box area was 6 (±2) square meters in size, with an open space of the same size, considering the principles of animal welfare, thus avoiding social stress due to collective manipulation. The animals were randomly assigned to two groups: control (CTR, n = 12) and treated (TRT, n = 13) groups. Both groups were fed with a commercial diet (Royal Canin, Supplementary material) from at least 7 days before the beginning of the study. The amount of daily food was calculated based on the following equation [17]:

ME (kcal/day) = 110 × kg BW 0.75

A placebo (maltodextrin powder) or a supplement containing *S. boulardii* (1 × 10^9^ CFU di/kg of feed) was added to the food of dogs belonging to the CTR or TRT group, respectively, once a day for 35 consecutive days.

### 2.2. Nutritional Parameters

Body weight (BW) was recorded at T0 and after 35 days (T5) days by the same veterinarian. Body condition score (BCS) is an effective assessment of body fat [18,19]; scores between 1 and 9 were assigned by the same trained veterinarian by visual examination and palpation of the animal at TO and T5. A score of 4 or 5 represents the ideal score. Feces were subjected to direct examination, and fecal score ranging from 1 to 7 (FS) was assigned at T0 and T5.

### 2.3. Laboratory Analysis

Fresh feces were collected by the breeder, in the morning, by using a sterile spatula and stored in a sterile plastic bag (box/dog code). They were then kept and transported at 4 °C to the laboratory. At the beginning of the study (T0) and after 7 (T1), 14 (T2), 21 (T3), 28 (T4), and 35 (T5) days, the following parameters on the fecal samples were calculated, as reported in the Supplementary Materials: calprotectin, lactoferrin, zonulin, histamine, cortisol, IgA, SCFA, and indole/skatole. The same technician performed the analysis following a blinded sample identification protocol. The DNA Extraction and Amplicon Target Sequencing procedures on fecal samples to determine the microbiota and mycobiota are reported in detail in the Supplementary Materials.

### 2.4. Statistical Analysis

The statistical analysis for the nutritional data and the laboratory data on fecal samples was performed by using IBM SPSS Statistics V27.0.0 software (Chicago, IL, USA). In relation to the nutritional parameters, a paired *t*-test was performed to see differences between the beginning and the end of the study for each treatment group.

The laboratory data were tested by fitting a generalized linear mixed model (GLM) that allowed the analytes to depend on linear predictors such as diet, time, and their interaction through a gamma probability distribution with a nonlinear link function (log). The animal was also included as a random effect to account for repeated measurements. A hybrid method for parameter estimation was used for both the GLMs, and a type III analysis with Wald chi-square test was applied to assess the model effects. All the obtained results were expressed as least-squares means and standard error of the mean (SEM), and the interactions between the factor levels were evaluated by pairwise contrasts. The *p*-values < 0.05 were considered statistically significant.

The sequencing data were analyzed by the Quantitative Insights into Microbial Ecology (QIIME) 2 [20]. A cutadapter was used for the filtering of primers and adapters. Sequencing denoising was performed by the DADA2 algorithm [21], removing low-quality bases, chimeric sequences, and sequences shorter than 300 bp by using the DADA2 denoise-paired plugin of QIIME2. Amplicon sequence variants (ASVs) were then used for taxonomic assignment, using the QIIME feature-classifier plugin against the Greengenes 16S rRNA gene database for the microbiota and the manually build database for the mycobiota [22]. Taxonomy assignment for 16S and 26S was double-checked on BLAST suite tools. QIIME2 diversity script was used to perform alpha and beta diversity analysis. Non-normally distributed variables were calculated as median (range interquartile). Metataxonomic variables were compared by the pairwise Kruskal test.

## 3. Results

All dogs remained healthy during the study, and no side effects (e.g., vomiting/diarrhea) were recorded. No food waste was found in any of the stalls throughout the period. There was no change in food consumption.

No difference in BW, BCS, and FS was recorded between T0 and T5 (*p* > 0.05) in each group.

**Figure 1:**
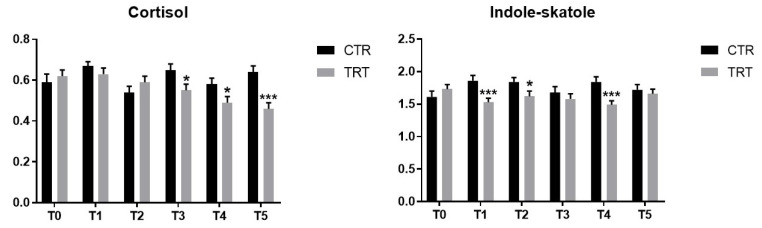
Concentration of cortisol (pg/mg) and indole/skatole (μmol/g) in the control (CRT) and treated (TRT) groups at each time point (T0 to T5). Graph bars with asterisks indicate significant differences between the dietary treatments within each sampling time; * = *p* < 0.05, and *** = *p* < 0.001.

At the beginning of the study (T0), the animals showed no significant differences (*p* > 0.05) for any of the fecal parameters analyzed (Table 1). *S. boulardii* supplementation had a significant effect on zonulin and indole/skatole (*p* < 0.05 and *p* < 0.001, respectively; Table 1). In particular, the TRT dogs showed lower concentration of fecal zonulin and indole/skatole when compared to the CTR group (*p* < 0.05 and *p* < 0.001, respectively; Table 1). However, a decrease in indole/skatole concentrations was observed at T1, T2, and T4 only (*p* < 0.05, Figure 1). Similarly, a significant diet*time interaction was identified for the fecal cortisol (*p* < 0.001, Table 1), with its concentrations decreasing at T3, T4, and T5 after the supplementation of *S. boulardii* (*p* < 0.05, Table 1). On the contrary, calprotectin was affected by time only (*p* < 0.001), with the lowest concentration at T5 (*p* < 0.001, Table 1). The other fecal parameters were not influenced by either of the considered variables (*p* > 0.05, Table 1).

The alpha diversity of microbiota and mycobiota did not show any significant difference between CRT and TRT groups (data not shown).

**Figure 2:**
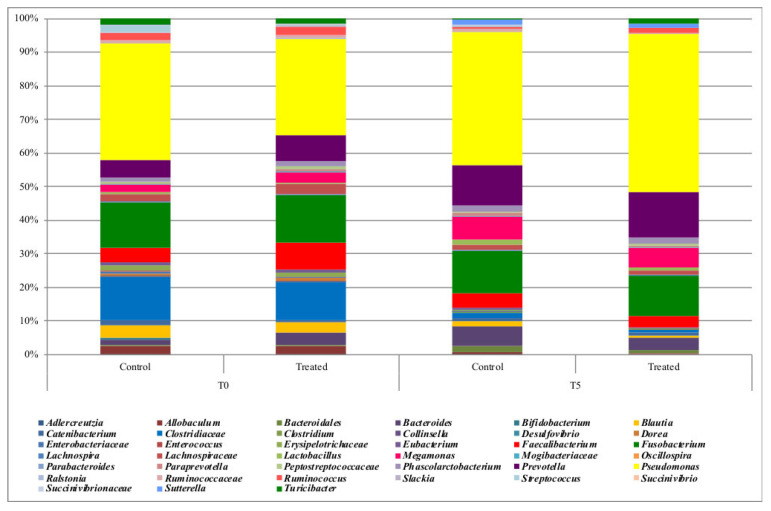
Relative frequency of the main bacterial ASVs in fecal samples of dogs fed with control or treated with probiotic during the trial. Graph bars indicate the 15 replicates per each sampling point.

**Figure 3:**
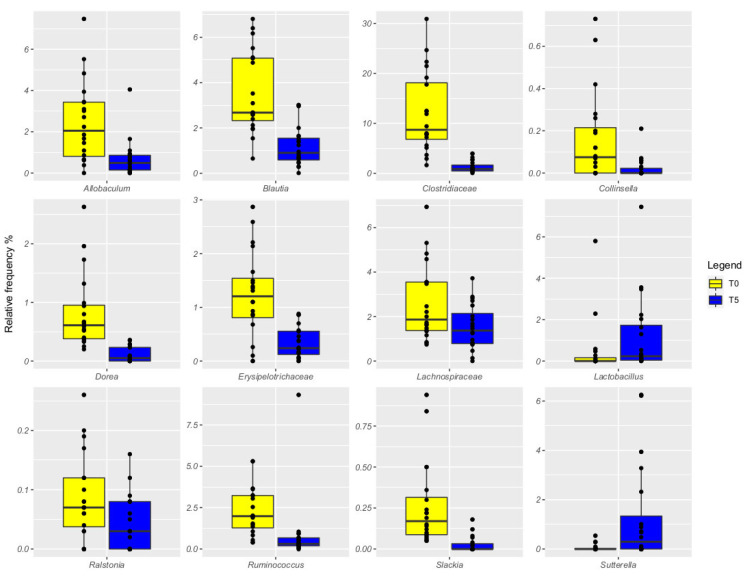
Relative frequency of differentially abundant bacterial ASVs in fecal samples of dogs during the experimental trial. Pairwise Kruskal–Wallis test, FDR < 0.05.

The CRT samples were dominated by *Pseudomonas* (35% and 40%, respectively, at T0 and T5), *Fusobacterium* remained constant across time (13%), *Clostridiaceae* decreased over time (12% and 1%, respectively), and *Prevotella* increased (from 5% to 12%, Figure 2). Dogs fed with the tested probiotic showed the presence of *Pseudomonas* at a relative frequency increasing from 28% at T0 to 46% at T5, *Clostridiaceae* decreasing from 11% at T0 to 1% at T5, and *Prevotella* increased from 7% to 13% at the end of the trial (Figure 3). When comparing the gut microbiota between T0 and T5, we observed that *Allobaculum*, *Blautia*, *Clostridiaceae*, *Dorea*, *Erysipelotrichaceae*, *Lachnospiraceae*, *Ralstonia*, *Ruminococcus*, and *Slackia* were more abundant at T0 compared to T5 in both groups (Figure 3).

By comparing the relative frequency between CRT and TRT groups, we did not observe any significant differences in the microbiota composition. However, we found that *Dorea* was the only one significantly affected by the probiotic administration at the end of the trial (FDR < 0.05), when data were compared to the CTR’s.

**Figure 4:**
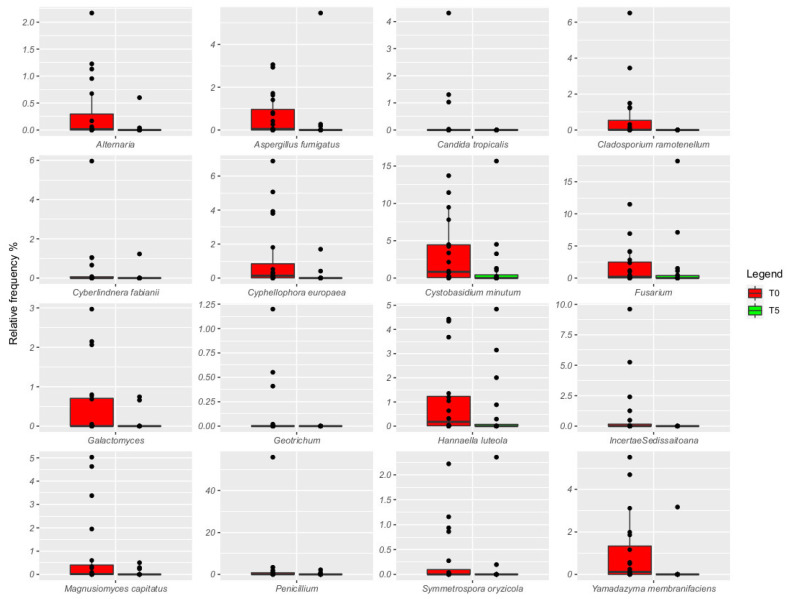
Relative frequency of differentially abundant fungal ASVs in fecal samples of dogs during the experimental trial. Pairwise Kruskal–Wallis test, FDR < 0.05.

**Figure 5:**
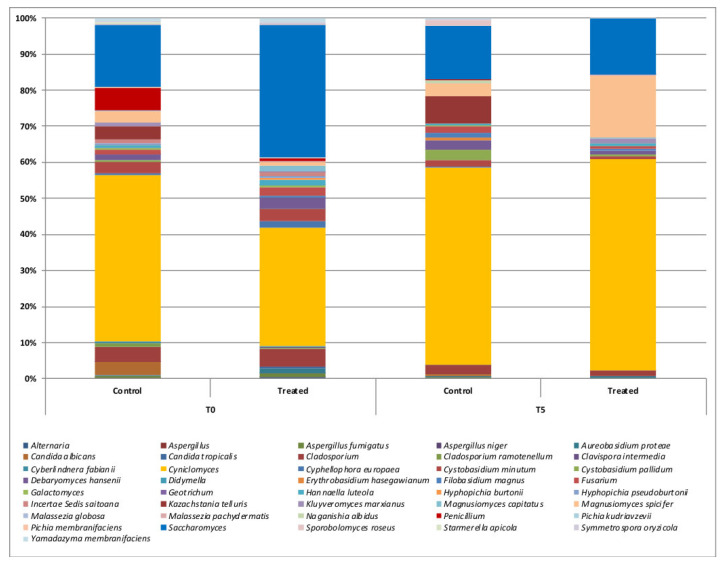
Relative frequency of the main fungal ASVs in fecal samples of dogs fed with control (C) or probiotic during the trial. Graph bars indicate the average of 15 fecal samples of dogs as replicate per each sampling point.

Regarding the mycobiota composition, *Clyniclomyces* was the most abundant in all samples (45% and 54% relative frequency in the CTR group, and 32% and 58% in the TRT at T0 and T5, respectively). *Saccharomyces* was more abundant in samples from the TRT dogs (about 35%) compared to the CRT (about 17%) at T0. At the end of the trial, the relative frequency decreased to 15% in both groups. *Penicillium* was found in the CTR group, with a frequency of 6% at T0 and 7% at T5. Its presence in the TRT group was less than 1% at both time points. *Cladosporium* was mostly present in probiotic samples at T5, reaching 17% (Figure 4). By comparing CTR and TRT, *Magnusiomyces capitatus* and *Malassezia pachydermatis* were the only two ASVs that were significantly associated with probiotic samples (Figure 5, FDR < 0.05). By comparing the relative frequency of fungi across time in both animal groups, we observed that T0 was characterized by the highest presence of *Alternaria*, *Aspergillus fumigatus*, *Cladosporium ramotenellum*, *Cyphellophora europaea*, *Cystobasidium minitum*, *Fusarium*, *Galactomyces*, *Hannaella luteola*, and *Yamadazyma membranicaciens* (Figure 5, FDR < 0.05).

## 4. Discussion

In recent years, changes in the gut microbiota have been found to be a critical determinant of host health [23]. The condition of intestinal eubiosis is very relevant for the psycho-physical well-being of an animal and can be put at risk by critical physiological status (weaning and aging) or life conditions, such as confined environment in farm or kennel. The recent literature shows probiotics as promising molecules to preserve intestinal health and to maintain the well-being of the organism. The use of probiotics has become promising for the treatment and prevention of various diseases in companion animals [1]. The aim of this study was to evaluate the efficacy of a diet supplemented with *S. boulardii*, evaluating the general health and the nutritional conditions of the animals. At the beginning of the experiment, all animals involved in our study were healthy, and there were no significant differences in all the parameters considered. The administration of *S. boulardii* did not cause any short-term adverse effects, as already reported by other authors [24]. There were no differences in BW and BCS in dogs treated with *S. boulardii* compared to the CTR group, suggesting that *S. boulardii* did not adversely affect these parameters and that animals ate the correct amount of food during the study.

Regarding the analysis of fecal parameters, lactoferrin is an iron-binding glycoprotein, and it is an important component of neutrophilic granulocytes; its concentration in the stool increases during intestinal inflammation as a result of the mucosal infiltration of leukocytes. In our study, lactoferrin did not vary in the two groups of dogs, meaning that there is no serious pathological state [25].

Zonulin is a 47 k Da protein released by several cell lines in the body, including epithelial cells lining the small intestine, that act on the intestinal tight junction [26]. In our study, we did not find significant differences between groups; therefore, the subjects did not show an increase in intestinal permeability. Short-chain fatty acids (SCFAs), mainly acetate, propionate, and butyrate, are primary end products of bacterial fermentation of non-digestible fiber foods. They have a regulatory effect on gastrointestinal motility and several beneficial effects on host health, including immunomodulatory effects in the intestine [27].

Indole/skatole and histamine have direct toxic effects on the intestinal mucosa. Putrefactive compounds also contribute to the nauseating smell typically associated with feces [28]. N-Methylhistamine (NMH), a product of histamine metabolism, is a proinflammatory biomarker of mast cell activation and degranulation. It can be measured in serum, urine, and stool samples [29]. The indole/skatole and N-Methylhistamine (NMH) analysis did not show significant differences in the two groups, thus indicating no negative effect of the supplement.

On the other hand, the supplementation with *S. boulardii* has produced positive effects on inflammatory markers (calprotectin), on the decrease of the immune response (IgA), and on psycho-physical stress (cortisol). Calprotectin and IgA have been suggested to be the non-invasive markers of canine intestinal health [30,31]. Our results showed that, at the end of the experiment, a significant reduction of calprotectin, cortisol, and IgA was found in the TRT group. These fecal biomarkers are relevant for the assessment of intestinal immunity or inflammation in dogs [31].

Calprotectin contributes to about 60% of the protein content of the neutrophil cytosol. Any disturbance of the mucosal architecture due to the inflammatory process causes the escape of neutrophils and, therefore, of calprotectin into the intestinal lumen and their subsequent excretion in the feces [32]. Other studies have reported a significant correlation between calprotectin levels and inflammatory states such as inflammatory bowel disease [33,34] or chronic inflammatory enteropathies [30,35]. Therefore, the decrease in fecal calprotectin levels assessed in dogs treated in our study could indicate a reduction in inflammation and a more stable intestinal environment, as also reported by Heilmann and colleagues (2018).

Secretory IgA is the most important humoral protective immune factor in the intestine. It inhibits adhesion, colonization, and microbial penetration, as well as the absorption of food antigen [36]. Our results showed an adjuvant effect on the mucosa of orally administered yeast. The gut microbiota and microbial metabolites are important for maintaining gut homeostasis. The decrease in IgA levels evaluated after the administration of *S. boulardii* indicates a lower immune reaction in the gut, and this can suggest a lower inflammatory status.

A wide range of stressors can induce the activation of the hypothalamus–pituitary–adrenal (HPA) axis with increased levels of glucocorticoids in the blood stream [37]. Among these molecules, cortisol is essential not only to cope with stressful conditions, but also for the proper functioning of the body and brain. It regulates numerous basal processes such as fat and glucose metabolism, blood pressure, and inflammatory and immune responses and aids in adaptation to environmental stress [38]. A recent research study has shown that the intestinal microbiota influences the physiological and cognitive functions of the brain and that, conversely, psychological stress negatively affects the GI function. Communication between intestinal bacteria and the central nervous system occurs through the enteric nervous system (ENS) and the endocrine, immune, and metabolic pathways [39,40]. Cortisol was found in several matrices, such as blood, saliva, hair, urine, and feces [41]. On farm animals, the use of fecal cortisol to assess stress levels over the long term in high-volume commercial breeding conditions was suggested by several authors [42]. In line with these studies, a lower production of cortisol could be correlated to a better ability of the animals to cope with the breeding environment [43,44]. A cortisol analysis performed on feces offer the advantage to collect samples in a non-invasive way, decreasing possible bias in the interpretation of the results due to the method of sampling [45]. In accordance with several reports on human responses related to the use of probiotics and fecal cortisol concentrations [46,47,48,49,50,51], our results showed a decrease in cortisol in this substrate, and we can suppose an improvement in the adaptive animal response to the environment and a decrease in stress levels when animals receive the integrated diet. Currently, a few studies regarding fecal cortisol concentrations in healthy dogs managed in domestic condition by owners have been published [52,53,54]. On the other hand, studies suggest that dogs in commercial breeding establishments or shelters showed an increased incidence of behavioral and emotional problems compared with dogs from other sources, especially noncommercial breeders. The literature shows that dogs’ cortisol levels in the high-volume commercial environment are still lacking. The possible causes of abnormal behaviors could be associated with distress [55,56]. In confined conditions, the environment limits the expression of dog species-specific behaviors. The potential sources of stress are related to inadequate socialization due to isolation or limited positive interactions with conspecifics and humans, confined environments with spatial restriction combined with lack of environmental stimuli, overcrowding of the boxes, competition for resources (food, resting area, etc.), and imbalances in hierarchies related to group revision in the same area [7,8,9]. In dogs, the persistent condition of stressful stimuli causes physical and psychological health problems, along with greater susceptibility to disease [6].

Dietary probiotic administration did not remarkably influence the gut microbiota of dogs in the present study, with the only exception of an increased abundance of *Dorea* being detected at the end of the trial [57]. This may be considered a positive finding, as *Dorea* usually manifests a reduced abundance in dogs with inflammatory bowel disease and other enteropathies [58]. The absence of a clear probiotic-related impact on the gut microbiota is partially in agreement with a recent study performed by Reference [59], wherein the inclusion of the probiotic alone (*Lactobacillus acidophilus*) had a minimal influence on most gut health outcomes, but more effects when administered along with prebiotics. Both CTR and TRT dogs displayed *Pseudomonas*, *Fusobacterium*, *Clostridiaceae*, and *Prevotella* as predominant members of their gut microbiota. As *Fusobacterium* is a commensal bacterium living in gut of healthy humans and dogs [60] and either *Clostridium* or *Prevotella* genera encounter SCFA-producing bacteria [5], this scenario suggests the identification of a healthy intestinal microbiota. However, an increased abundance of *Pseudomonas* has frequently been observed in dogs with chronic intestinal inflammation [58], thus representing a potential negative finding. However, the ability of *Pseudomonas* to produce GABA from glutamate has recently made this taxon an interesting marker to differentiate healthy dogs from epileptic dogs, as the latter are characterized by a significantly reduced abundance of *Pseudomonas* in their gut microbiota [61] Finally, several taxa resulted in being increased in both groups at the end of the experiment, thus confirming the role of the dog’s age as one of the most important intrinsic factors affecting the intestinal microbiota [62].

Gut mycobiota are not often studied in humans or animals since they represent 1–2% of the total microbiome, and often fungi are transient commensal of the GI tract. However, gut fungi can have beneficial effects in the host due to their ability to modulate metabolism such as nutrient extraction, vitamin production, and defense against pathogens [63,64,65]. A dog’s gut mycobiota are not often studied, and it was already reported that the class *Saccharomycetes* is the core taxa identified in healthy and diseased animals, followed by *Wickerhamomycetaceae*, *Pleosporaceae*, *Schizothyriaceae*, and *Trichocomaceae* [66]. At the genus level, the most commonly observed taxa belong to *Pichia*, *Cryptococcus*, *Candida*, and *Trichosporon* [67].

Here, we observed the predominance of *Clyniclomyces.* This taxon is usually associated with the GI of rabbits, where it is unclear if this organism causes or is a co-cause of diarrhea [68]. Studies inferred a potential correlation between *Clyniclomyces* and disease status of dogs; however, its predominance can be considered a clinically non-significant finding [68]. *Saccharomyces* was associated with dogs belonging to the TRT group, and it is a common constituent of the human and animal mycobiota, with several anti-inflammatory proprieties [69,70]. It has to be pointed out that sequences of the D1/D2 domain of the 26S rDNA are identical in both species [71]. *Penicillium* and *Cladosporium* are also components of the dog’s gut [72]. *Penicillium* is often associated with mice fed with a high-fat diet [73], while *Cladosporium* is most commonly identified in healthy dogs [67]. *Malassezia* is the major component of the fungal skin microbiota of mammals; however, its role in maintaining gut health is still not clear [74].

We observed a shift of several fungi across time, but not related to the administration of the tested probiotic. In particular, we observed a reduction of several taxa that are a common constituent of the gut mycobiota across time.

## 5. Conclusions

This research confirms the beneficial effects of *S. boulardii* on dog gut health. The administration of probiotics was well tolerated by the animals and showed positive effects on some fecal parameters. The interest of the scientific community in *S. boulardii* is relatively recent in both human and veterinary medicine. The results of this study showed that *S. boulardii* could be used to counter intestinal inflammation and psycho-physical stress in animals. Further studies are needed to understand the effects on animal health over a longer period of time and on different age groups and breeds

## Figures and Tables

**Table 1:** Nutritional parameters and laboratory analytes of the dogs depending on the group (G) they belong to (CRT = control; TRT = treated), time (T), and their interaction (G × T).

	Group (G)		Time (T)						SEM		*p*-Value		
	CTR	TRT	T0	T1	T2	T3	T4	T5	G	T	G	T	G × T
Laboratory analytes (unit)													
pH	6.51	6.50	6.50	6.54	6.50	6.52	6.46	6.50	0.06	0.05	0.982	0.152	0.161
Calprotectin (µg/g)	5.95	5.57	5.99 ^ab^	6.04 ^a^	5.94 ^b^	5.63 ^cd^	5.64 ^c^	5.32 ^d^	0.85	0.60	0.753	<0.001	0.108
Lactoferrin (µg/g)	1.53	1.32	1.45	1.45	1.31	1.38	1.49	1.44	0.22	0.16	0.489	0.260	0.330
Zonulin (ng/mL)	52.51	50.36	49.58	52.35	49.84	53.79	50.18	52.96	0.77	1.16	0.046	0.250	0.710
Cortisol (pg/mg)	0.61	0.55	0.60	0.65	0.57	0.60	0.53	0.54	0.02	0.02	0.090	0.100	<0.001
Immunoglobulin A (mg/g)	47.71	48.17	48.87	48.68	48.33	47.40	47.66	46.75	1.70	1.23	0.849	0.100	0.116
Short chain fatty acids (μmol/g)	143.56	146.96	148.11	145.94	139.55	146.24	145.04	146.77	21.39	15.54	0.912	0.112	0.180
Indole/skatole (μmol/g)	1.76	1.60	1.67	1.73	1.63	1.66	1.69	1.68	0.04	0.06	<0.001	0.937	0.001

Means with superscript letters (^a, b, c, d^) identify significant differences among the sampling times (*p* < 0.05).

## Data Availability

Data are available upon request to the authors.

## Supplementary Materials

The following supporting information can be downloaded at: https://www.mdpi.com/article/10.3390/vetsci9080389/s1, Information on fecal parameters and DNA extraction and amplicon target sequencing [22,75,76].
