# Supplementary material for: Effects of Saccharomyces boulardii Supplementation on Nutritional Status, Fecal Parameters, Microbiota, and Mycobiota in Breeding Adult Dogs
Source: Vet Sci. 2022 Jul 28;9(8):389. doi: 10.3390/vetsci9080389 (PMC9414249; doi:10.3390/vetsci9080389)
Supplement: Supplementary file 1 [file vetsci-09-00389-s001.zip › vetsci-1792293-supplementary.pdf]

Effects of *Saccharomyces boulardii* supplementation on nutritional status, fecal parameters, microbiota and mycobiota in breeding adult dogs

Giorgia Meineri [1], Elisa Martello [2*], David Atuahene [1], Silvia Miretti [1], Bruno Stefanon [3], Misa Sandri [3], Ilaria Biasato [4], Maria Rita Corvaglia [4], Ilario Ferrocino [4], Luca Simone Cocolin [4]

1 Department of Veterinary Sciences, School of Agriculture and Veterinary Medicine, University of Turin, 10095, Grugliasco (TO), Italy. giorgia.meineri@unito.it, david.atuahene@unito.it, silvia.miretti@unito.it,

2 Centre for Evidence Based Healthcare, School of Medicine, University of Nottingham, NG5 1PB, Nottingham, UK. martello.elisa@gmail.com

3 Department of Agrifood, Environmental and Animal Science, University of Udine, 33100 Udine, Italy. bruno.stefanon@uniud.it, misa.sandri@nutrigenefood.com

4 Department of Agricultural, Forest and Food Sciences, University of Turin, 10095, Grugliasco (TO), Italy. ilaria.biasato@unito.it, ilario.ferrocino@unito.it, lucasimone.cocolin@unito.it, mariarita.corvaglia@unito.it

*Correspondence: martello.elisa@gmail.com

Supplementary material

Fecal parameters

<u>Calprotectin</u>

Fecal calprotectin concentration was determined via a species - specific enzyme - linked immunosorbent assay (ELISA) developed and analytically validated at the Gastrointestinal Laboratory at Texas A&M University. Spot fecal samples (1.0 ± 0.3 g) were collected and stored frozen (–20 °C) for 2–20 months until analysis. Fecal samples were thawed and extracted, and biomarker concentrations were measured in 2 batches of all specimens using an ELISA kit (Bühlmann Laboratories AG, Schönenbuch, Switzerland). Aliquots of approximately 100 mg of faces were homogenized in 5 mL of extraction buffer according to the manufactured instruction. Two mL of the homogenate was then centrifuged in a microcentrifuge for 5 minutes at 250 rpm. Then 3,000 g plus 100 μL of the diluted supernatant (1:50 with incubation buffer) were incubated at room temperature 25 °C and placed into a microtiter plate coated with a monoclonal capture antibody highly specific to the calprotectin heterodimeric and polymeric complexes. After incubation for three days, samples were washed with a phosphate saline solution (Ph 8) and then incubated. A further washing step with a phosphate saline solution (Ph 8) was performed and an aliquot of tetramethylbenzidine (uL 5) was added to stop the reaction. The optical density was immediately measured at 450nm using a microplate spectrophotometer reader.

<u>Lactoferrin</u>

Fecal lactoferrin was determined with a specific ELISA kit (IBD-SCAN; TechLab, Blacksburg, VA). Sterile water (300μl) was added to each solid fecal sample, mixed thoroughly and then centrifuged for 2 minutes at 1500g to sediment particulates. Aliquots of 100 μl were used to perform the assay. Each sample and a control were prepared in duplicate, adding 100μl of solution to well strips. Phosphate Buffered Saline (pH 8) was used as negative control. Balance solution (10μl) was added to each well containing the fecal sample supernatant. 50μl of enzyme conjugate was added to each well, except blank controls. The plate was placed on a vortex machine for 30 seconds to mix the contents thoroughly. The plate was sealed and incubated at 37 ° C for 1 hour and then wells were washed. The wells were refilled with wash solution and washed a total of five times. Plates were inverted and the wells emptied of contents by firmly tapping onto blotting paper to dry. To all the empty wells, including blank control well, 50 μl of Substrate A and 50 μl of Substrate B was added. Plate was sealed and incubated in the dark at 37 ° C. After 15 minutes, 50 μl of stop solution was added to each well, including blank control wells to stop the reaction and the contents were mixed thoroughly.

Zonulin

Zonulin concentration was determined by using an ELISA kit (Immundiagnostik AG). The assay used the competitive binding technique. Biotinylated zonulin tracer was added to the samples, standards, and positive and negative controls as a competitor to the sample's own zonulin. The intensity of the color was inversely proportional to the zonulin concentration in the sample. Samples were read using a 450 nm microplate spectrophotometer reader.

Imunoglobuline A.

Capture enzyme-linked immunosorbent assays (ELISAs) was used to quantify the concentration of IgA in faces. Polyvinyl microtiter plates (Sigma-Aldrich) were coated overnight at 4 ° C with 100 μl of the primary antibody diluted in carbonate-bicarbonate buffer (0.05 M; pH 9.6). Residual protein binding was blocked with 200 μl of 20% defatted milk at 37 ° C for 1 h. All incubations were carried out in sealed humidified chamber, and plates were washed three times with a buffer (PBST) between incubations. Purified IgA as well as pooled serum and bile samples, were used in the assessment of ELISA specificity. Doubling dilutions of each sample (75 μl) in PBST were made on each plate from a starting dilution of 1:50. All samples were fully titrated together with the standard on each plate. Starting dilutions for the standards were 1: 2,000 (IgA). The plates were incubated overnight at 5 ° C prior to addition of the secondary and tertiary antibodies (75 μl), which were both incubated for 2 h at room temperature.

N - methylhistamine (NMH).

NMH was measured by using stable isotope dilution gas chromatography (GCMS). Briefly, 50 pg of trideuterated NMH was added as an internal standard to 200 μL of each fecal extract (diluition 1:5). Then 200 μL of sodium borate buffer (pH 9, 10 mM) was added. The sample was vortexed and then applied to a solid-phase silica extraction column. The columns were washed with changes of chromatographygrade water, and then the sample was eluted by using 0.1 N HCl acidified methanol. The eluted samples were placed in a heating block and evaporated to dryness by using nitrogen. The dried sample was reconstituted with 300 μL of 20% methanol in chloroform before application to the second solid-phase silica extraction column. The column was washed with 150 μL of 20% methanol in chloroform. The sample was eluted by using four 1-mL volumes of methanol: chloroform: ammonium hydroxide (25: 25: 1, v / v) and dried as described earlier. Derivitization was achieved by adding 200 μL ethyl acetate, 40 μL pyridine and 100 μL pentafluoropropionic anhydride to the sample and incubating at 64 ° C for 40 min. In the next partitioning step, 500 μL of 0.5 M Tris buffer was added to each sample, followed by 1.5 mL of hexane. The samples were vortexed for 1 min and centrifuged at 574 × g for 1 min. The hexane (upper) layer was collected, another 1.5 mL of hexane was added to each sample, and the process was repeated. The 2 hexane fractions were combined and evaporated to dryness. Before its transfer to a gas chromatography – MS autosampler vial, the residue was reconstituted with 30 μL of ethyl acetate and vortexed. The gas chromatography – MS analysis was performed by using a gas chromatograph and mass selective detector with a dimethylpolysiloxance capillary column; all other conditions (temperature, carrier gas, gradient, and pressure) used were similar to what has been described in the earlier fecal NMH assay in dogs. A standard curve from 0 to 5000 pg / μL was processed prior to each run, to evaluate assay performance. NMH and deuterated isotopes were quantified by using the ions at an m / z of 417 and 420, respectively. Fecal concentrations of NMH were backcalculated for the wet weight of the sample and reported as nanograms per gram of faces.

Putrefactive Fecal Compounds (Indole, Skatole)

Indole and skatole were extracted according to [1]. Briefly, 2 g of faces were mixed with 5 mL of methanol, covered with parafilm, and incubated for 1 h at 4°C with frequent mixing. After centrifuging at 2,124 × g for 10 min at 4°C the supernatant was removed and 5 mL of methanol was added to the pellet, mixed thoroughly and kept for 1 h, as described previously by [1]. A combination of both supernatants was then analyzed by gas chromatography for indole and skatole using a gas chromatograph. The internal standard was 5- chloro indole. Initial temperature of the inlet was 200°C and injection by splitless mode. Initial temperature of the oven was 85 ° C maintained for 2.0 min, and the temperature program included an increase by 10 ° C per min until 250 ° C maintained for 4.0 min. The carrier gas was helium and the FID (Flame-Ionization Detector) temperature was 220 ° C.

Short Chain Fatty Acids (SCFA)

Concentrations of SCFAs (i.e. acetate, propionate, and butyrate) in faces were measured using a stable isotope dilution gas chromatography-mass spectrometry (GC-MS) assay. Briefly, the fecal samples were weighed, diluted 1: 5 in extraction solution (2N hydrochloric acid, HCl), and frozen at −80 ° C until analysis (up to 3 months). After thawing, samples were homogenized by a multitube vortexer for 30 minutes at room temperature (RT), and then fecal suspensions were centrifuged for 20 minutes at 2100g and 4C. Supernatants were then collected using serum filters. From each sample, 500 µL of supernatant was mixed with 10 µL of internal standard (200 mM heptadeuterated butyric acid) and extracted using a C18 solid phase extraction column. Samples were derivatized using N-tert-butyldimethylsilyl-Nmethyltrifluoroacetamide (MTBSTFA) at RT for 60 minutes. A GC coupled with an electron ionization MS was used for chromatographic separation and quantification of the derivatized samples. Separation was achieved using a DB1 ms capillary column. The GC temperature program was as follows: 40C held for 0.1 minute, increased to 70C at 5C / min, 70C held for 3.5 minutes, increased to 160C at 20C / min, and finally increased to 280C at 35C / min, then held for 3 minutes. The total run time was 20.53 minutes. The MS was operated in electron impact positive-ion mode with selective ion monitoring at mass-to-charge ratios (M / Z) of 117 (acetate), 131 (propionate), 145 (butyrate), and 152 (heptadeuterated butyrate; internal standard). Quantification was based on the ratio of the area under the curve of the internal standard and each of the fatty acids. The lower detection limits of fecal concentrations of acetate, propionate, and butyrate were 1.33, 0.43, and 0.12 µmol / g, respectively. Samples 66 with fecal SCFA concentrations less than the lower detection limits of acetate, propionate, and butyrate were considered as 1.32, 0.42, and 0.11 µmol / g, respectively. To take into account the difference in water content between fecal samples, final concentrations of fecal SCFAs were adjusted by fecal dry matter (DM) and expressed as µmol / g of fecal DM.

Cortisol

A cortisol Enzyme Immuno Assay (EIA) was used to analyze fecal extracts. The assay employed a cortisol-horseradish peroxidase ligand and antiserum (No. R4866; CJ Munro, University of California, Davis, CA) and cortisol standards (hydrocortisone; Sigma-Aldrich, St. Louis, MO). The polyclonal antiserum was raised in rabbits against cortisol-3-carboxymethyloxime linked to bovine serum albumin and cross-reacts with cortisol 100%, prednisolone 9.9%, prednisone 6.3%, cortisone 5% and <1% with corticosterone, desoxycorticosterone, 21-desoxycortisone, testosterone, androstenedione, androsterone, and 11-desoxycortisol. The EIA was performed in 96-well microtiter plates (Nunc-Immuno, Maxisorp Surface; Fisher Scientific, Pittsburgh, PA) coated 14–18 h previously with cortisol antiserum (50 µl / well; diluted 1: 20,000 in coating buffer; 0.05 M $NaHCO_3$, pH 9.6). Fecal extracts evaporated to dryness and diluted in steroid buffer (0.1 M $NaPO_4$, 0.149 M NaCl, pH 7.0) were assayed in duplicate. Cortisol standards (50 µl, range 3.9-1000 pg / well, diluted in assay buffer, 0.1 M $NaPO_4$, 0.149 M NaCl, 0.1% bovine serum albumin, pH 7.0) and sample (50 µl) were combined with cortisol-horseradish peroxidase (50 µl, 1: 8500 dilutions in assay buffer). Following incubation at room temperature for 1 h, plates were washed five times before 100 µl substrate buffer [0.4 mM 2,2'-azino-di- (3-ethylbenzthiazoline sulfonic acid) diammonium salt, 1.6 mM H_2O_2, 0.05 M citrate, pH 4.0] was added to each well. After incubation on a shaker for 10–15 min, the absorbance was measured at 405 nm. Parallel displacement curves were obtained for each species by comparing serial dilutions of pooled fecal extracts and the cortisol standard preparation. Intra- and interassay coefficients of variation were 6.4% (n = 26 replicates of a single sample) and 11.0% (n = 57 assays), respectively. Assay sensitivity was 3.9 pg / well at maximum binding.

DNA Extraction and Amplicon Target Sequencing

Total DNAs from fecal samples (200 mg) were extracted using the RNeasy Power Microbiome KIT (Qiagen, Milan, Italy) following the manufacturer's instructions. RNase (5ng/mL) was added to digest RNA in the DNA samples, after an incubation of 1 h at 37 °C. DNA was quantified using the QUBIT dsDNA Assay kit (Life Technologies, Milan, Italy) and standardized at 5 ng/µL. One µl of each DNA suspension was used as template for PCR amplification by using primers 16SR and 16SF spanning the V3-V4 region of the 16S rRNA gene following the procedure described by [2]. Fungi were detected by amplifying the D1 domain of the 26S rRNA gene[3]. Library preparation and sequencing by MiSeq instrument (Illumina, San Diego, CA) were carried out according to the manufacturing instructions.

Chemical analysis of the administered food

Table S1. Composition of the dry food (Royal Canin) administered to all the animals included in the study.

Protein	22%
Fat	18%
Ash	6.2%
Fiber	1.8%
Carbohydrates	52%
Vitamin A	22.400 UI
Vitamin D3	1000 UI
Iron	43mg /kg
Iodine	4,3 mg /Kg
Brass	15 mg/kg
Magnesium	56 mg/kg
Zinc	168 mg /Kg
Selenium	0,11 mg/kg

References

22. Flickinger, E., et al., *Nutrient digestibilities, microbial populations, and protein catabolites as affected by fructan supplementation of dog diets.* Journal of animal science, 2003. **81**(8): p. 2008-2018.

75. Klindworth, A., et al., *Evaluation of general 16S ribosomal RNA gene PCR primers for classical and next-generation sequencing-based diversity studies.* Nucleic acids research, 2013. **41**(1): p. e1-e1.

76. Mota-Gutierrez, J., et al., *Metataxonomic comparison between internal transcribed spacer and 26S ribosomal large subunit (LSU) rDNA gene.* International journal of food microbiology, 2019. **290**: p. 132-140.
